# Sex differences in the levels of key mitochondrial markers in subjects with mild cognitive impairments and Alzheimer’s disease

**DOI:** 10.1192/j.eurpsy.2025.1678

**Published:** 2025-08-26

**Authors:** B. Bigio, R. Lima-Filho, O. Barnhill, F. Sudo, C. Drummond, N. Assuncao, B. Vanderborght, J. Beasley, S. Young, A. Korman, D. Jones, D. Sultzer, S. Ferreira, P. Mattos, E. Head, F. Tovar-Moll, F. De Felice, M. Lourenco, C. Nasca

**Affiliations:** 1 Psychiatry, NYU Grossman School of Medicine, New York, United States; 2 Institute of Medical Biochemistry Leopoldo de Meis, Rio de Janeiro, Brazil; 3 Rockefeller University, New York, United States; 4 D’Or Institute for Research and Education (IDOR), Rio de Janeiro, Brazil; 5 Duke University , Durham, United States; 6 Duke University , Durham, Brazil; 7 NYU Grossman School of Medicine, New York; 8 University of California, Irvine, Irvine; 9 D’Or Institute for Research and Education (IDOR), Rio de Janeiro, United States; 10 Queen’s University, Kingston, Canada; 11 Neuroscience & Physiology , NYU Grossman School of Medicine, New York, United States

## Abstract

**Introduction:**

The lack of accessible plasma biomarkers to identify target populations limits the promise of precision medicine for Alzheimer’s Disease (AD). Amnestic mild cognitive impairment (aMCI) is an important risk for AD and often occurs years before the onset of AD.

**Objectives:**

Based on an emerging mechanistic model of mitochondrial mechanisms of brain plasticity, we studied the role of acetylcarnitine and free-carnitine levels assayed in plasma as potential markers of cognitive dysfunction in subjects with aMCI or early-AD.

**Methods:**

We used available samples from two independent cohorts well characterized for clinical and neuropsychological characteristics together with ultraperformance liquid chromatography-tandem mass spectrometry and computational approaches. Cerebrospinal fluid (CSF) measures of b-amyloid accumulation and t-Tau levels were also available and used in computational modeling.

**Results:**

Within the primary cohort, our data showed decreased levels of carnitine in relation to cognitive function as assessed by using the Mini Mental Status Exam (MMSE) in women but not men with CI as compared to age- and sex-matched HC. Furthermore, the magnitude of carnitine deficiency reflected the severity of cognitive dysfunction in a sex-specific manner (women: p = 0.015; men: p = 0.441). Our data also replicated the prior finding of decreased LAC levels in both women and men with AD, supporting the robustness of the study samples assayed in our new study. Using computational approaches, we found that the integration of these mitochondrial measures with canonical CSF biomarkers improves diagnostic accuracy. A second cohort provides a validation of the sex-specific relationship between free-carnitine deficiency and the severity of cognitive dysfunction.

**Conclusions:**

Taken together with prior mechanistic studies in rodents, the current findings support future research on the development of individualized treatment models targeting sex-specific changes in mitochondrial metabolism.

**Disclosure of Interest:**

None Declared

